# Trends in the use of thyroid diagnostics and treatments between 2008 and 2019 in Germany

**DOI:** 10.1038/s41598-024-77896-4

**Published:** 2024-11-04

**Authors:** Arulmani Thiyagarajan, Niklas Koenen, Till Ittermann, Henry Völzke, Ulrike Haug

**Affiliations:** 1 Department of Clinical Epidemiology, Leibniz Institute for Prevention Research and Epidemiology – BIPS, Bremen, Germany; 2 Department of Biometry and Data Management, Leibniz Institute for Prevention Research and Epidemiology – BIPS, Bremen, Germany; 3 Faculty of Mathematics and Computer Science, University of Bremen, Bremen, Germany; 4 Institute for Community Medicine, University Medicine Greifswald, Greifswald, Germany; 5 Faculty of Human and Health Sciences, University of Bremen, Bremen, Germany

**Keywords:** Thyroid diagnostics, Thyroid treatments, Age-specific prevalence, Age-standardized prevalence, Germany, Endocrinology, Health care

## Abstract

Following implementation of a national prevention program against iodine deficiency-related disorders in 1993 in Germany, the prevalence of thyroid disorders declined, but aggregated drug dispensation data suggested a recent increase in thyroid hormone use. We aimed to systematically investigate trends in the use of thyroid diagnostic and treatment measures between 2008 and 2019 in Germany. Using the GePaRD database (claims data from ~ 20% of the German population), we determined the annual age-specific and age-standardized prevalence of the use of thyroid diagnostics (thyroid-stimulating hormone (TSH) measurement, thyroid ultrasound, scintiscan, biopsy) and treatment (medication, surgery, radioiodine therapy) from 2008 to 2019 stratified by sex. Between 2008 and 2019, the age-standardized prevalence of TSH measurement increased by 36% in males (from 165 to 225 per 1,000 persons) and by 24% in females (from 295 to 367 per 1,000). The prevalence of thyroid ultrasound use showed no clear time trend, while there was a decrease in the prevalence of scintiscan use (males/females: decrease by 42/43%) and biopsy (males/females: decrease by 42/34%). The prevalence of levothyroxine use increased by ~ 31% both in males (from 36 to 47 per 1,000) and females (from 134 to 175 per 1,000), while the prevalence of thionamide use, iodine use, thyroidectomy, and radioiodine therapy decreased. Our study suggests a continued decline in the prevalence of clinically significant thyroid disorders between 2008 and 2019. The substantial increase in the prevalence of TSH measurement and levothyroxine use during this time period might indicate overuse of diagnostics and overtreatment, warranting further investigation.

**Supplementary Information:**

The online version contains supplementary material available at 10.1038/s41598-024-77896-4.

## Introduction

Until the early 1990s, Germany was an area with mild-to-moderate iodine deficiency due to its low natural occurrence of iodine in the soil. In 1993, an optimized nationwide iodine fortification program for table salt and cattle food was implemented. Several indicators supported the effectiveness of this program, such as an elevated iodine status in the general population, reduced goiter prevalence in schoolchildren, and a general decrease in the prevalence of iodine-deficient disorders (goiter, thyroid nodules, and hyperthyroidism in adults)^1–6^.

However, aggregated data on drug dispensation in Germany showed a marked increase in the prescription of thyroid hormones between 2006 and 2016. In 2021, levothyroxine ranked among the five most frequently prescribed drugs in Germany^7,8^. Since the trend does not match the overall improvement in the iodine status of the German population, the question arises whether it may be partly explained by overtreatment or overuse of diagnostics. A comprehensive analysis of individual-level data on the use of thyroid diagnostics and treatment in the German population would be helpful to explore this potential explanation, but such a study has not been conducted so far.

To fill this gap, we aimed (1) to characterize persons using thyroid diagnostics or treatment regarding age, sex and healthcare utilization and (2) to determine the annual prevalence of thyroid diagnostics and treatment use between 2008 and 2019 overall and stratified by sex and age based on a large German claims database comprising ~ 20% of the German population.

## Materials and methods

### Data source

We used the German Pharmacoepidemiological Research Database (GePaRD), which is based on claims data from four statutory health insurance providers and currently includes information on about 25 million persons who have been insured with one of the providers since 2004 or later^9,10^. In addition to demographic data, GePaRD comprises information on the dispensation of prescription drugs in the outpatient setting as well as on outpatient (i.e., from general practitioners and specialists) and inpatient services and diagnoses. Specific information on thyroid diagnostics and treatment is obtained based on codes of the German uniform assessment standard (“*Einheitlicher Bewertungsmaßstab*”; EBM), the operations and procedures coding system (“*Operationen- und Prozedurenschlüssel”*; OPS) and the Anatomic Therapeutic Chemical (ATC) classification. Per data year, there is information on ~ 20% of the general German population, and all geographical regions of Germany are represented. In Germany, about 90% of the general population are covered by statutory health insurances^11^. Core characteristics of the German health insurance system are uniform access to all levels of care and a free choice of providers.

The use of GePaRD data for this study was approved by all four health insurance providers as well as the German Federal Office for Social Security and the Senator for Health, Women and Consumer Protection in Bremen as their responsible authorities. Informed consent for studies based on claims data is required by law unless obtaining consent appears unacceptable and would bias results, which was the case in this study. According to the Ethics Committee of the University of Bremen studies based on GePaRD are exempt from institutional board review. The study was performed according to the institutional guidelines of the Leibniz Institute for Prevention Research and Epidemiology – BIPS. All data was analyzed anonymously and the authors did not have access to identifying information.

## Study design and study population

We conducted cross-sectional analyses for each calendar year between 2008 and 2019. For each year, we considered all persons with a continuous insurance coverage of 365 days (a gap of ≤ 30 days was allowed) in the respective year. We excluded those with no or no consistent information on sex or age, as well as those residing outside of Germany.

We determined the annual prevalence separately for the following thyroid diagnostic procedures (A) and treatments (B): A1) thyroid-stimulating hormone (TSH) measurement, A2) thyroid ultrasound, A3) scintiscan, A4) biopsy, B1) levothyroxine, B2) iodine, B3) thionamides, B4) thyroidectomy and B5) radioiodine therapy. The number of persons with at least one billing code (EBM/OPS/ATC) of the respective category in the respective year was used as the numerator of the prevalence (code list is shown in Additional file 1 - Supplementary Table 1). The number of all included persons in the respective year was used as the denominator. For TSH measurement, we also determined the annual prevalence of persons who had 1, 2, 3, 4, or ≥ 5 TSH measurements, respectively, in the calendar year.

### Data analysis

We characterized men and women with at least one thyroid diagnostic procedure or treatment exemplified by the year 2019 (the most recent data year available for analysis) regarding the distribution of age (grouped into: ≤14, 15 to 29, 30 to 44, 45 to 59, 60 to 74, and ≥ 75 years) and the frequency of healthcare utilization. Healthcare utilization was assessed based on the total number of different outpatient treatment episodes in 2019.

For each calendar year, we calculated age-specific and age-standardized prevalences per 1,000 persons stratified by sex for the use of thyroid diagnostics and treatments as described above. For age standardization, we used the age distribution of the German population in 2019 as a reference^12^. In addition, we determined the age-specific and age-standardized prevalences stratified by sex and healthcare utilization, with healthcare utilization categorized by the quintiles of the number of outpatient treatment episodes.

We performed all analyses using SAS 9.4 (SAS Institute, Inc., Cary, North Carolina, USA).

## Results

### Characteristics of persons with thyroid diagnostics/treatment in 2019

We identified 5,074,512 persons with at least one thyroid diagnostic procedure and 1,435,780 persons with at least one thyroid treatment in 2019. The majority of these persons were female (diagnostic procedures: 65%; treatments: 73%) and ≥ 45 years (diagnostic procedures: 66%, treatments: 83%; Table 1). The median number of outpatient treatment episodes in 2019 as a measure of healthcare utilization was 13 in females and 11–12 in males. The characteristics of persons with at least one thyroid diagnostic procedure or treatment in other calendar years (2008–2018) were similar to those in 2019 (data not shown).

**Table 1:** Characteristics of persons who had at least one thyroid diagnostic procedure or thyroid treatment in 2019.

Characteristics	Thyroid diagnostic procedure ^a^		Thyroid treatment ^b^	
	Male (*n* = 1,752,110)	Female (*n* = 3,322,402)	Male (*n* = 383,936)	Female (*n* = 1,051,844)
Age				
Mean ± SD	53.4 ± 20.6	52.7 ± 19.7	61.4 ± 19.6	63.0 ± 18.1
Median [IQR]	56 [38–69]	54 [37–68]	66 [50–77]	66 [52–77]
Age groups, n (column-%)				
≤ 14 years	76,138 (4.4)	71,474 (2.2)	4,697 (1.2)	4,883 (0.5)
15 to 29 years	192,708 (11.0)	394,115 (11.9)	31,463 (8.2)	60,824 (5.8)
30 to 44 years	299,474 (17.1)	693,109 (20.9)	41,849 (10.9)	103,294 (9.8)
45 to 59 years	437,327 (24.9)	868,631 (26.1)	69,795 (18.2)	224,786 (21.4)
60 to 74 years	432,136 (24.7)	753,671 (22.7)	115,485 (30.1)	323,747 (30.8)
≥ 75 years	314,327 (17.9)	541,402 (16.3)	120,647 (31.4)	334,310 (31.8)
Frequency of healthcare utilization^c^				
Mean ± SD	12.1 ± 7.1	14.2 ± 7.3	13.4 ± 7.5	14.4 ± 7.4
Median [IQR]	11 [7–16]	13 [9–18]	12 [8–18]	13 [9–18]

SD - Standard deviation; IQR - Interquartile range.

^a^ thyroid diagnostic procedure includes thyroid stimulating hormone measurement, ultrasound, scintiscan, and biopsy.

^b^ thyroid treatment includes levothyroxine, thionamide, iodine, thyroid surgery, and radioiodine therapy.

^c^ Healthcare utilization is calculated based on the total number of different outpatient treatment episodes in 2019, irrespective of whether or not they were related to thyroid disease.

### Trends in the use of thyroid diagnostic procedures

**Fig. 1:**
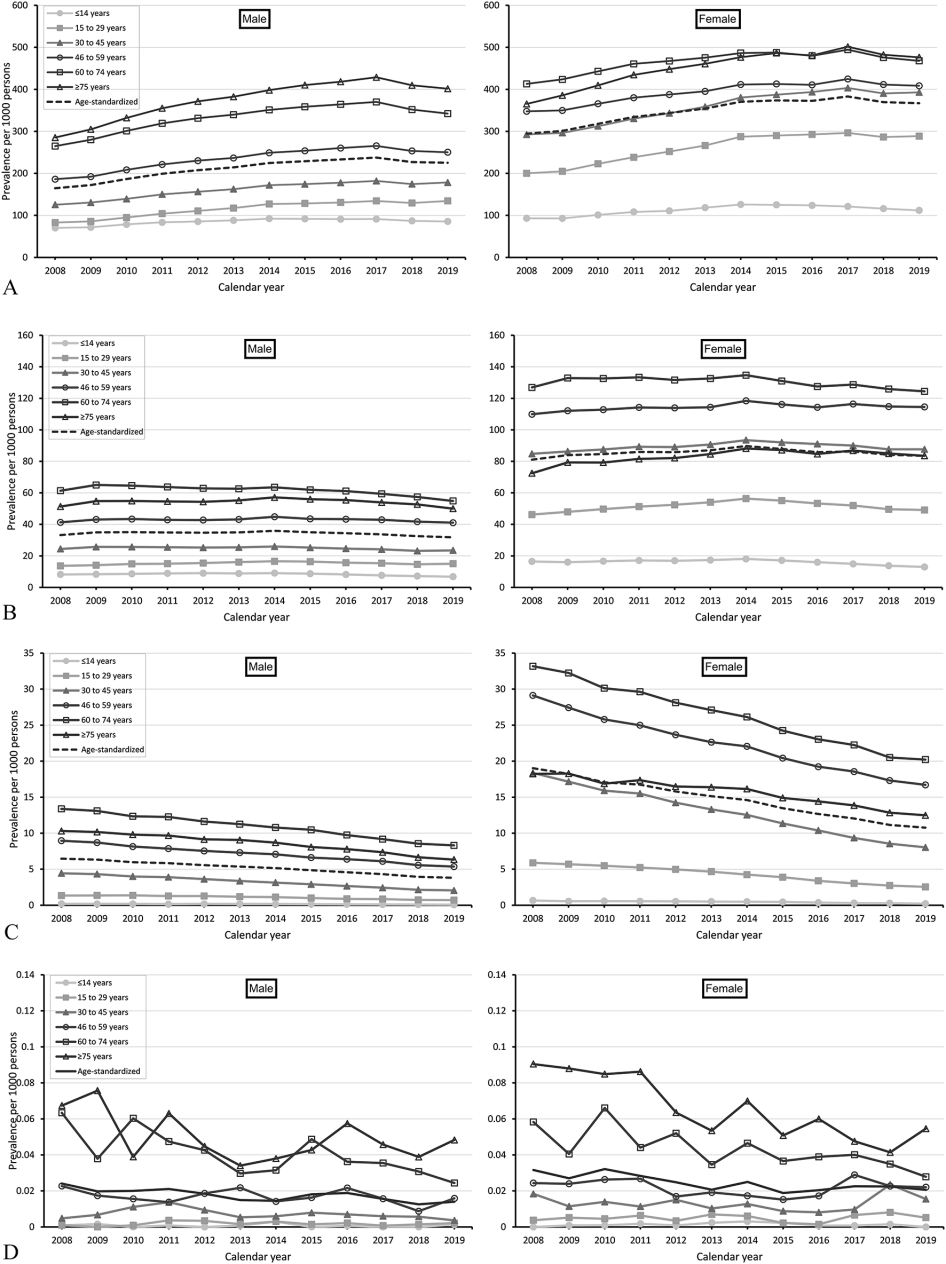
(**A**) Annual age-standardized and age-specific prevalence of thyroid-stimulating hormone measurement per 1,000 persons from 2008 to 2019, stratified by sex (left: male, right: female). (**B**) Annual age-standardized and age-specific prevalence of thyroid ultrasound per 1,000 persons from 2008 to 2019, stratified by sex (left: male, right: female). (**C**) Annual age-standardized and age-specific prevalence of thyroid scintiscan per 1,000 persons from 2008 to 2019, stratified by sex (left: male, right: female). (**D**) Annual age-standardized and age-specific prevalence of thyroid biopsy per 1,000 persons from 2008 to 2019, stratified by sex (left: male, right: female).

Figure 1A, B and C, and D show the annual age-specific and age-standardized prevalences (per 1,000 persons) of TSH measurement, thyroid ultrasound, scintiscan, and biopsy, respectively, for the years 2008 to 2019. The prevalences increased with age and were about two to three times higher in females than in males in most age groups.

For most diagnostic procedures, the annual prevalence changed over time. The age-standardized prevalence (per 1,000 persons) of at least one TSH measurement increased between 2008 and 2017 from 164.6 to 237.8 in males and from 294.7 to 383.0 in females (relative increase: males: 44%, females: 30%). In 2019, the prevalence was 225.3 in males and 367.0 in females (Fig. 1A). In absolute terms, the largest increase was observed among those aged ≥ 75 years (males: from 285.3 to 428.7, females: from 365.3 to 501.5; relative increase: males: 50%, females: 37%). Analyses stratified by the number of TSH measurements per person (i.e., persons with 1, 2, 3, 4, and ≥ 5 TSH measurements) showed that the age-standardized prevalences increased over time for each category (See Additional file 1 - Supplementary Fig. 1.1 to 1.5).

The annual use of thyroid ultrasound (i.e., both the age-specific and age-standardized prevalences) showed no clear time trend. From 2008 to 2019, the age-standardized prevalence per 1,000 persons ranged between 31.8 and 35.9 in males and 81.0 and 89.7 in females (Fig. 1B). The annual use of thyroid scintiscan and biopsy decreased in both males and females. From 2008 to 2019, the age-standardized prevalence of scintiscan (per 1,000 persons) decreased from 6.5 to 3.8 in males and 19.0 to 10.8 in females (relative decrease: males: 42%, females: 43%; Fig. 1C). In absolute terms, the decline was strongest among those aged between 60 and 74 years (males: 13.4 to 8.3, females: 33.2 to 20.2; relative decrease: males: 38%, females: 39%). For thyroid biopsy, the age-standardized prevalence (per 1,000 persons) decreased from 0.024 to 0.014 in males and 0.032 to 0.021 in females (relative decrease: males: 42%, females: 34%; Fig. 1D).

## Trends in the use of thyroid treatment

**Fig. 2:**
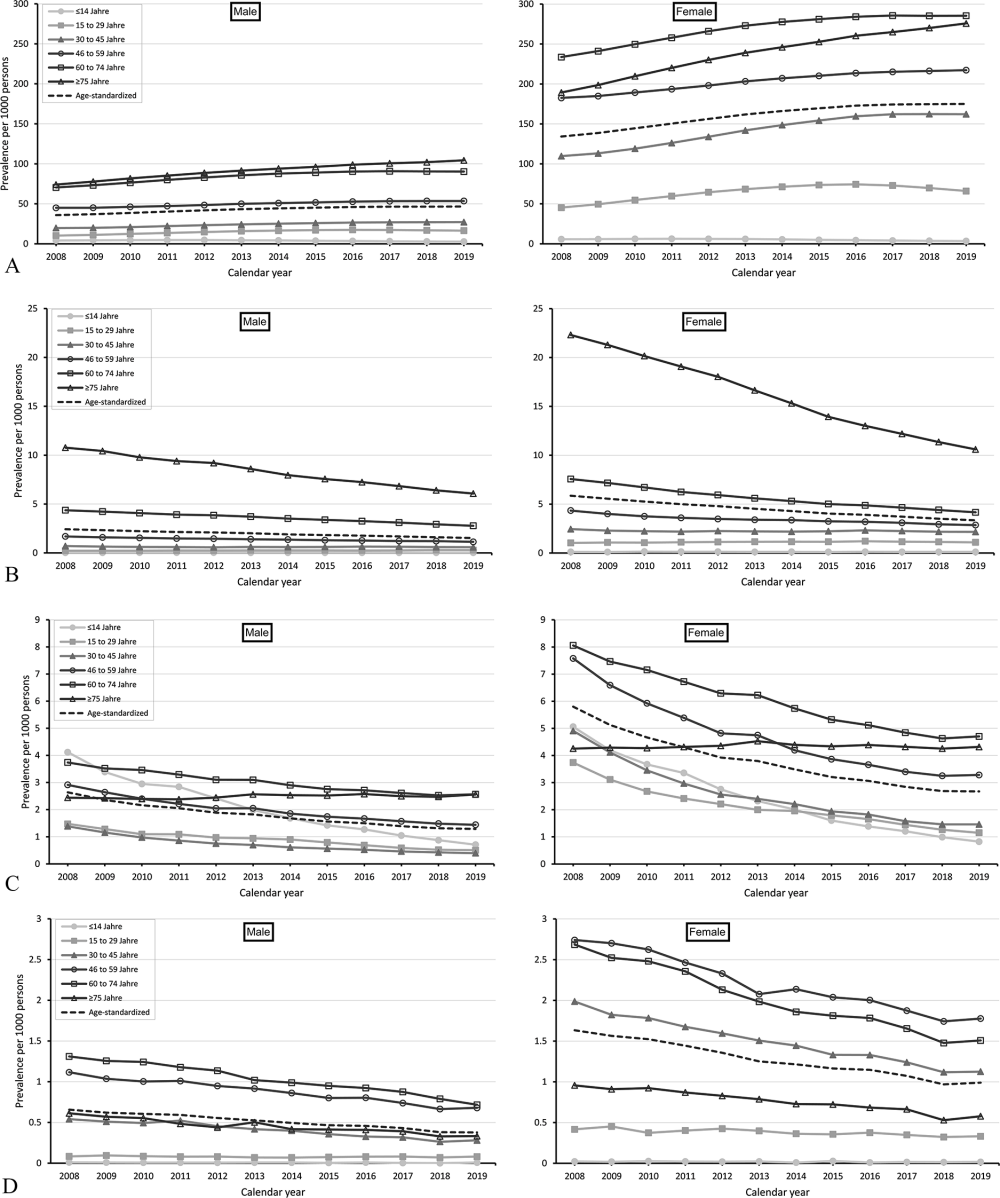

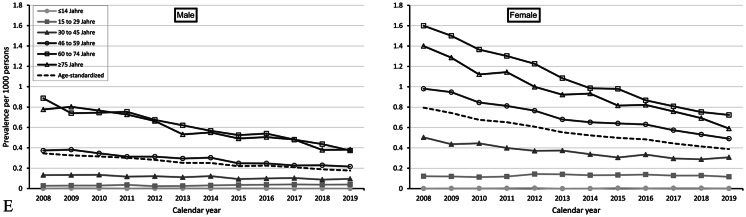
(**A**) Annual age-standardized and age-specific prevalence of levothyroxine use per 1,000 persons from 2008 to 2019, stratified by sex (left: male, right: female). (**B**) Annual age-standardized and age-specific prevalences of thionamide use per 1,000 persons from 2008 to 2019, stratified by sex (left: male, right: female). (**C**) Annual age-standardized and age-specific prevalence of iodine use per 1,000 persons from 2008 to 2019, stratified by sex (left: male, right: female). (**D**) Annual age-standardized and age-specific prevalence of thyroidectomy per 1,000 persons from 2008 to 2019, stratified by sex (left: male, right: female). (**E**) Annual age-standardized and age-specific prevalence of radioiodine therapy per 1,000 persons from 2008 to 2019, stratified by sex (left: male, right: female).

Figure 2A, B, C and D, and 2E show time trends in the annual age-specific and age-standardized prevalences (per 1,000 persons) of levothyroxine, thionamides, iodine, thyroidectomy, and radioiodine therapy, respectively, from 2008 to 2019. Among thyroid treatments, levothyroxine was most frequently used, while radioiodine therapy was least frequently used. For all thyroid treatments, the prevalence increased with age and was markedly higher (about two to four times higher) in females than in males.

For all thyroid treatments, the prevalence changed over time. The annual use of levothyroxine increased in males and females. From 2008 to 2019, the age-standardized prevalence (per 1,000 persons) of levothyroxine use increased from 35.9 to 46.6 in males and from 134.3 to 175.1 in females (relative increase: males: 30%, females: 30%; Fig. 2A). In absolute terms, the largest increase was observed in those aged ≥ 75 years (males: 74.0 to 104.3, females: 189.3 to 275.8; relative increase: males: 41%, females: 46%).

In contrast, the prevalence of thionamide use, iodine use, thyroidectomy, and radioiodine therapy decreased in males and females. From 2008 to 2019, the age-standardized prevalence (per 1,000 persons) of thionamide use decreased from 2.4 to 1.5 in males and from 5.9 to 3.3 in females (relative decrease: males: 38%, females: 45%; Fig. 2B). In absolute terms, the largest decrease was observed in those aged ≥ 75 years (males: 10.8 to 6.1, females: 22.3 to 10.6) (relative decrease: males: 44%, females: 53%). For iodine, the age-standardized prevalence (per 1,000 persons) decreased from 2.6 to 1.3 in males and from 5.8 to 2.7 in females (relative decrease: males: 57%, females: 53%; Fig. 2C). In absolute terms, the largest decrease was observed in those aged ≤ 14 years (males: 4.1 to 0.7, females: 5.1 to 0.8; relative decrease: males: 83%, females: 84%). For thyroidectomy, the age-standardized prevalence (per 1,000 persons) decreased from 0.7 to 0.4 in males and from 1.6 to 1.0 in females (relative decrease: males: 43%, females: 38%; Fig. 2D). In absolute terms, the largest decrease was observed in those aged 60 to 74 years (males: 0.7 to 1.3, females: 2.7 to 1.5; relative decrease: males: 45%, females: 44%). For radioiodine therapy, the age-standardized prevalence (per 1,000 persons) decreased from 0.4 to 0.2 in males and from 0.8 to 0.4 in females (relative decrease: males: 50%, females: 50%; Fig. 2E). In absolute terms, the largest decrease was observed in those aged between 60 and 74 years (males: 0.9 to 0.4, females: 1.6 to 0.7; relative decrease: males: 55%, females: 56%).

## Correlation with healthcare utilization

As shown in Additional file 1 - Supplementary Fig. 2.1 to 2.8, for most thyroid diagnostics and treatments, the prevalence correlated with the frequency of healthcare utilization, particularly in women. For TSH measurement and levothyroxine use, for example, there was a linear increase in prevalence already between the bottom and the third quintile of the number of outpatient treatment episodes. For procedures such as thyroidectomy, the prevalence only increased after the third quintile in men, while in women, it also increased between the second and the third quintile.

## Discussion

To the best of our knowledge, this is the first study providing detailed information on the trends in the annual use of thyroid diagnostics and treatment by sex and age in Germany. Between 2008 and 2019, in both males and females, we observed that the annual prevalence of TSH measurements and levothyroxine use substantially increased. In contrast, the annual prevalence of thyroid scintiscan use, biopsy, thionamide use, iodine use, thyroidectomy, and radioiodine therapy decreased. Thyroid ultrasound use showed no clear time trend but varied with age and sex. The prevalence of all considered thyroid diagnostic procedures and treatments was markedly higher (up to four times) in females than males and in older persons; further, it showed a positive correlation with healthcare utilization.

The American Thyroid Association recommends a TSH measurement as an initial screening test to examine thyroid dysfunction among adults (≥ 35 years), and it further highlights the need for more frequent screening (i.e., done as a part of routine examination) among symptomatic and high-risk individuals (persons ≥ 60 years, with cardiovascular diseases, other systemic/ chronic diseases, family history)^13–15^. In addition, further diagnostic procedures such as thyroid ultrasound, scintiscan, and biopsy are necessitated if any abnormality is found in the clinical examination. The aging population in Germany^16^ is expected to lead to an increase in the absolute number of TSH measurements, but we calculated age-standardized and age-specific prevalences, i.e., the increasing trend observed in our study cannot be explained by these demographic changes. Also, the reasons for the substantial increase in the prevalence of TSH measurements among females aged between 30 and 45 years are unclear. Clinical trials investigating the treatment of subclinical hypothyroidism during early pregnancy on cognitive outcomes in children, might have drawn attention to this topic^17,18^. Even though these studies did not find an effect, they might still have led to an increase in the use of thyroid diagnostics and treatment in pregnant women or women planning a pregnancy.

Several studies examined the prevalence of thyroid disorders in Germany^2,19–23^, but there is a lack of data on the use of thyroid diagnostic procedures. Only one study from Mecklenburg–West Pomerania (a federal state in eastern Germany) using data from 2008 to 2012 examined the frequency of thyroid diagnostic procedures in the general population and reported that 30% (1,626/5,552) of them received at least one TSH measurement per year, 6.8% (378/5,552) at least one thyroid ultrasound, and 2.6% (146/5,552) at least one scintiscan^24^. While these findings are in line with the non-sex-specific prevalences in our study (Additional file 1 - Supplementary Fig. 3 to 6), we could not compare the sex- and age-specific findings nor the trends over time, as such results were not provided by this study. Regarding findings from other countries, a study from Italy examining the cross-sectional trends (per 1,000 persons) in the prevalence of thyroid diagnostic procedures from 2010 to 2017 also reported an increase in the prevalence of TSH measurement, but this trend was less pronounced than in our study (males: from 90 to 104, females: from 235 to 252)^25^. Also, the prevalence of TSH measurement and ultrasound use observed in our study were almost twice as high as in the Italian study.

In our study, the annual prevalence of levothyroxine use substantially increased between 2008 and 2019 in males and females, while the corresponding prevalence of thionamide use and other treatments decreased (including in non-sex-specific prevalences, see Additional File 1 – Supplementary Fig. 7 to 11). The latter supports existing evidence from cohort studies collecting high-quality primary data which showed that the prevalence of clinically significant thyroid disorders in general, including structural anomalies, has reduced substantially after the introduction of the iodine fortification program in Germany^2^. There were also studies reporting a shift of the TSH levels towards the right in response to this program^26^, similar to studies from several other countries showing increased rather than decreased TSH population levels a few years after improved iodine supply^27^. Using fixed cut-off levels for TSH values, i.e., not taking into account these changes due to the improved iodine supply, may lead to overdiagnosis and overtreatment of subclinical thyroid disease. The substantial increase in the prevalence of levothyroxine use may thus be, at least partly, a consequence of the increase in TSH measurements in combination with the current cut-off levels. Also, a study from Denmark using registry data reported that after implementation of the iodine fortification program, the incident use of thyroid hormone increased. It reached a steady state after 10 years but was above the level at the time of program implementation^28^. However, when comparing both countries, it has to be considered that the fortification program in Denmark was implemented on a mandatory basis and had a sharper increase in the iodine status compared to Germany^29^. Apart from potential overdiagnosis and overtreatment as a consequence of iodine fortification programs in combination with fixed cut-off levels, a study from the United States highlighted the possibility of overdiagnosing thyroid dysfunction when age-and race-specific TSH range is not employed^30^.

In our study, we observed markedly higher prevalences of TSH measurement and levothyroxine therapy in females than in males. Although this was partly expected due to the female-to-male ratio of the genetic predisposition towards thyroid dysfunction^31^, the extent of the difference in prevalences was still surprising. It might indicate that there is more overdiagnosis and overtreatment in women than in men. This is supported by the sex-specific patterns we observed for the correlation between healthcare utilization and the use of thyroid diagnostics and treatment although it should be noted that part of the increased healthcare utilization could also be due to a thyroid disease. Interestingly, also the increase in the incidence of thyroid cancers in Germany, which is assumed to be attributable to overdiagnosis, was more pronounced in women than in men^32^.

Even though there are plausible reasons supporting the hypothesis that there is an overdiagnosis and overtreatment of hypothyroidism in Germany, it still has to be mentioned that this hypothesis remains to be confirmed and that there could also be other reasons. In some countries including Denmark, an iodine-induced increase in the incidence of hypothyroidism following the fortification program was proposed, but this was only in the early years and plateaued afterwards^33^. So it is unlikely that the increasing trend observed during our study period in Germany is explained by such a mechanism.

Regarding strengths, our study benefits from a data source covering about 20% of the German population^10^. It enabled us to examine age and sex-specific trends over 11 years (from 2008 to 2019) and is free of non-responder and recall bias. Furthermore, it has been shown that the drug prescriptions in GePaRD are representative of the general German population and all persons with statutory health insurance in Germany, respectively^34,35^. There are also some limitations that should be kept in mind. First, claims data are generally limited with respect to information on medications dispensed in the hospitals. However, since most thyroid medications are prescribed in the outpatient setting, we do not think this has affected the trends. Second, claims data do not contain information on the indication of drugs, the results of TSH measurement, nor whether TSH measurement / an imaging procedure was done for routine screening, monitoring of an existing condition, or as follow-up test to clinical examination. This information would be interesting to better understand the reasons for the trends in the prevalence of TSH measurement and potential mechanisms of overuse of diagnostics. A longitudinal analysis describing patient journeys at an individual level based on claims data could also be informative in this regard and is planned as a next step, following this study consisting of year-wise cross-sectional analyses, which provided a general overview. Furthermore, an analysis focusing on the use of thyroid diagnostics and treatment before and during pregnancy is planned.

## Conclusions

In conclusion, our study suggests a continued decline in the prevalence of clinically significant thyroid disorders between 2008 and 2019. The substantial increase in the prevalence of TSH measurement and levothyroxine use during this time period might indicate overuse of diagnostics and overtreatment, warranting further investigation.

## Electronic Supplementary Material

Below is the link to the electronic supplementary material.

Supplementary Material 1

## Data Availability

As we are not the owners of the data we are not legally entitled to grant access to the data of the German Pharmacoepidemiological Research Database (GePaRD). In accordance with German data protection regulations, access to the data is granted only to employees of the Leibniz Institute for Prevention Research and Epidemiology – BIPS on the BIPS premises and in the context of approved research projects. The data that support the findings of this study are available from BIPS, but restrictions apply to the availability of these data, which were used under license for the current study, and so are not publicly available. Data are however available from the authors upon reasonable request and with permission of BIPS. The data protection concept of GePaRD must be followed. Please contact the corresponding author (haug@leibniz-bips.de) for inquiries related to the data used in this study.

## Abbreviations

ATC: Anatomic Therapeutic Chemical
EBM: Einheitlicher Bewertungsmaßstab
GePaRD: German Pharmacoepidemiological Research Database
OPS: Operationen- und Prozedurenschlüssel
TSH: Thyroid stimulating hormone
