# Author Correction: Trends in the use of thyroid diagnostics and treatments between 2008 and 2019 in Germany

**DOI:** 10.1038/s41598-025-02916-w

**Published:** 2025-05-27

**Authors:** Arulmani Thiyagarajan, Niklas Koenen, Till Ittermann, Henry Völzke, Ulrike Haug

**Affiliations:** 1 Department of Clinical Epidemiology, Leibniz Institute for Prevention Research and Epidemiology – BIPS, Bremen, Germany; 2 Department of Biometry and Data Management, Leibniz Institute for Prevention Research and Epidemiology – BIPS, Bremen, Germany; 3 Faculty of Mathematics and Computer Science, University of Bremen, Bremen, Germany; 4 Institute for Community Medicine, University Medicine Greifswald, Greifswald, Germany; 5 Faculty of Human and Health Sciences, University of Bremen, Bremen, Germany

Correction to: *Scientific Reports* 10.1038/s41598-024-77896-4, published online 04 November 2024

The original version of this Article contained errors in the Abstract.

“Between 2008 and 2019, the age-standardized prevalence of TSH measurement increased by 44% in males (from 165 to 238 per 1,000 persons) and by 31% in females (from 134 to 176 per 1,000).”

now reads:

“Between 2008 and 2019, the age-standardized prevalence of TSH measurement increased by 36% in males (from 165 to 225 per 1,000 persons) and by 24% in females (from 295 to 367 per 1,000).”

The original Article has been corrected.

