# Supplementary material for: Trends in the use of thyroid diagnostics and treatments between 2008 and 2019 in Germany
Source: Sci Rep. 2024 Nov 4;14:26710. doi: 10.1038/s41598-024-77896-4 (PMC11535039; doi:10.1038/s41598-024-77896-4)

Supplementary tables and figures of Trends in the use of thyroid diagnostics and treatment between 2008 and 2019 in Germany

Arulmani Thiyagarajan, Niklas Koenen, Till Ittermann, Henry Völzke, Ulrike Haug

Contents:

Table 1: Billing codes of thyroid diagnostics and treatments

Figure 1.1 to 1.5: Annual age-standardized and age-specific prevalence of persons with 1, 2, 3, 4, and ≥5 thyroid stimulating hormone measurement per 1,000 persons from 2008 to 2019, stratified by sex

Figure 2.1 to 2.8: Age-standardized and age-specific prevalence of thyroid stimulating hormone measurement, thyroid ultrasound, scintiscan, biopsy, levothyroxine, iodine, thyroidectomy, and radioiodine therapy per 1,000 persons in 2019, stratified by healthcare utilization

Figure 3: Annual age-standardized and age-specific prevalence of thyroid-stimulating hormone measurement per 1,000 persons from 2008 to 2019

Figure 4: Annual age-standardized and age-specific prevalence of thyroid ultrasound per 1,000 persons from 2008 to 2019

Figure 5: Annual age-standardized and age-specific prevalence of thyroid scintiscan per 1,000 persons from 2008 to 2019

Figure 6: Annual age-standardized and age-specific prevalence of thyroid biopsy per 1,000 persons from 2008 to 2019

Figure 7: Annual age-standardized and age-specific prevalence of levothyroxine use per 1,000 persons from 2008 to 2019 (male and female combined)

Figure 8: Annual age-standardized and age-specific prevalence of thionamide use per 1,000 persons from 2008 to 2019 (male and female combined)

Figure 9: Annual age-standardized and age-specific prevalence of iodine use per 1,000 persons from 2008 to 2019 (male and female combined)

Figure 10: Annual age-standardized and age-specific prevalence of thyroid surgery per 1,000 persons from 2008 to 2019 (male and female combined)

Figure 11: Annual age-standardized and age-specific prevalence of radioiodine therapy per 1,000 persons from 2008 to 2019 (male and female combined)

Table 1: Billing codes of thyroid diagnostics and treatments

	Corresponding billing code		
	EBM	OPS	ATC
**Thyroid diagnostic procedure**			
Thyroid-stimulating hormone measurement	32101		
Ultrasound	33012	3-003.1	
Scintigraphy	17320	3-701	
Biopsy – thyroid tissue		1-406.2, 1-407.2, 1-582.0, 1-859.0	
**Thyroid treatment**			
Radioiodine therapy	17370	8-530.0, 8-531	
Thyroidectomy		5-063, 5-061, 5-062, 5-064, 5-069.00, 5-069.10	
Levothyroxine (including combinations)			H03AA01, H03AA03, H03AA51, H03AA53,
Iodine			H03CA
Thionamide derivatives			H03BB

EBM: *Einheitlicher Bewertungsmaßstab* (German uniform assessment standard);
OPS: *Operationen- und Prozedurenschlüssel* (Operations and procedures coding);
ATC: Anatomical Therapeutic Chemical classification;

Figure 1.1: Annual age-standardized and age-specific prevalence of persons with 1 thyroid stimulating hormone measurement per 1,000 persons from 2008 to 2019, stratified by sex

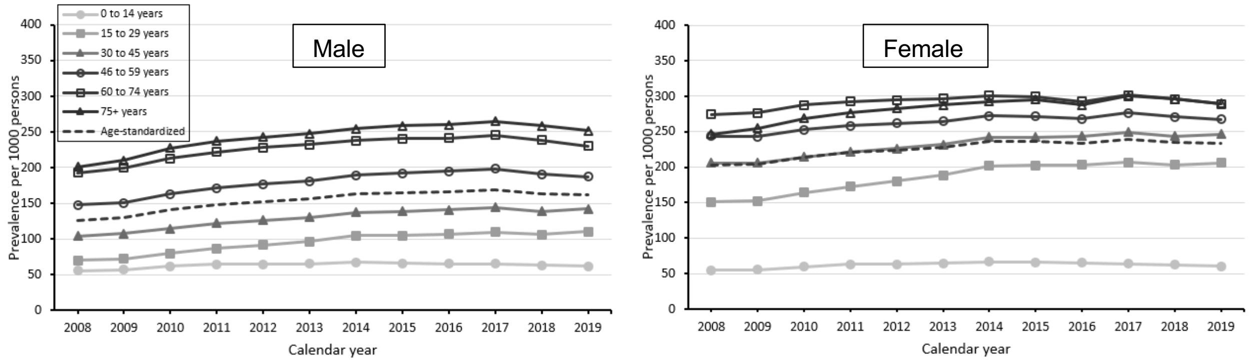

Figure 1.2: Annual age-standardized and age-specific prevalence of persons with 2 thyroid-stimulating hormone measurements per 1,000 persons from 2008 to 2019, stratified by sex

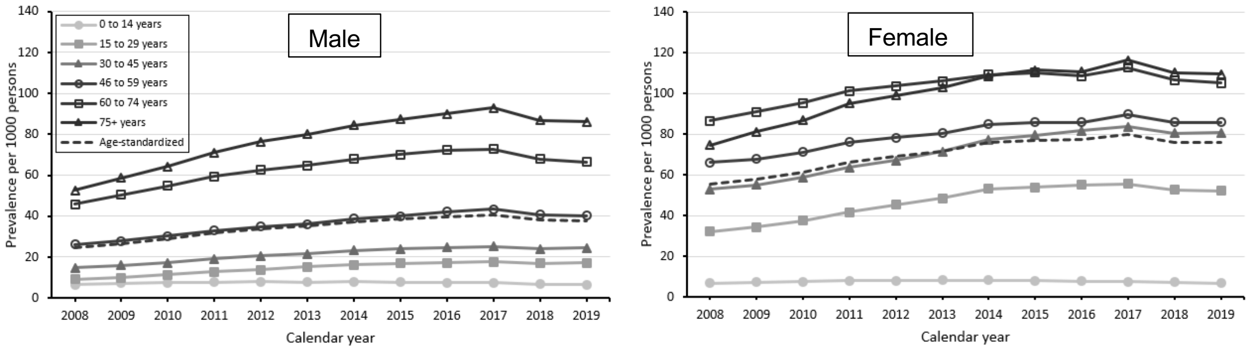

Figure 1.3: Annual age-standardized and age-specific prevalence of persons with 3 thyroid-stimulating hormone measurements per 1,000 persons from 2008 to 2019, stratified by sex

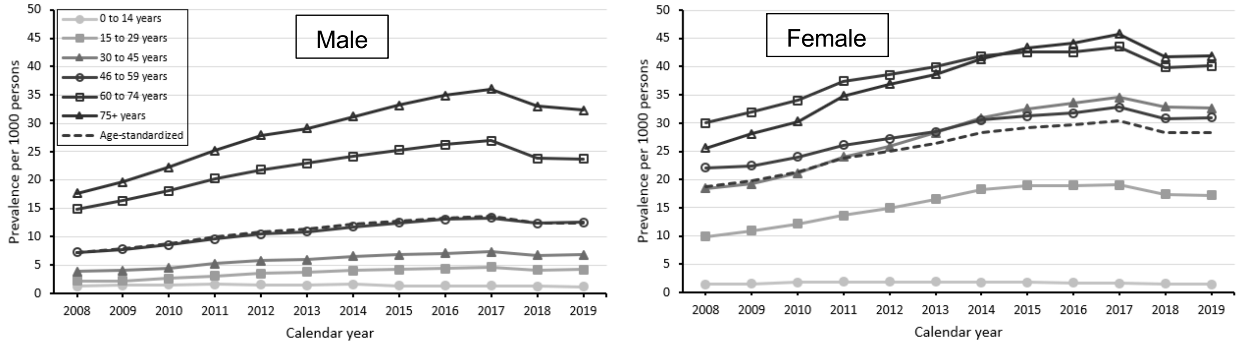

Figure 1.4: Annual age-standardized and age-specific prevalence of persons with 4 thyroid-stimulating hormone measurements per 1,000 persons from 2008 to 2019, stratified by sex

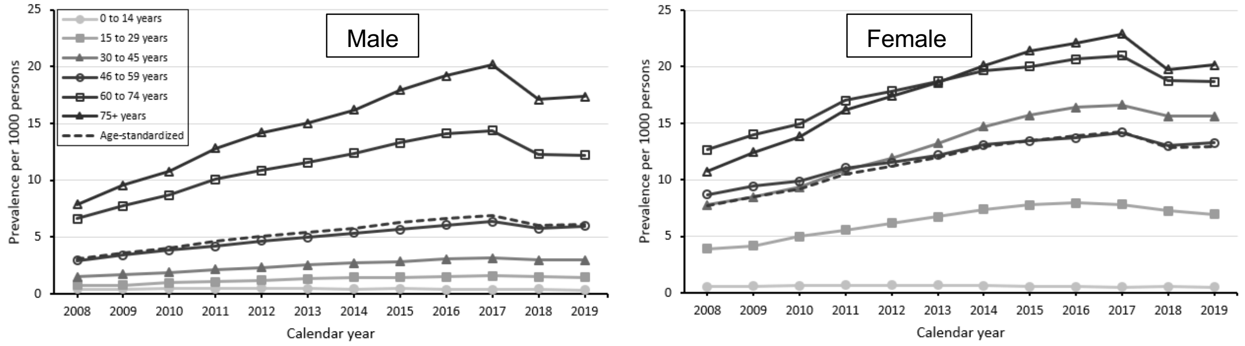

Figure 1.5: Annual age-standardized and age-specific prevalence of persons with ≥5 thyroid-stimulating hormone measurements per 1,000 persons from 2008 to 2019, stratified by sex

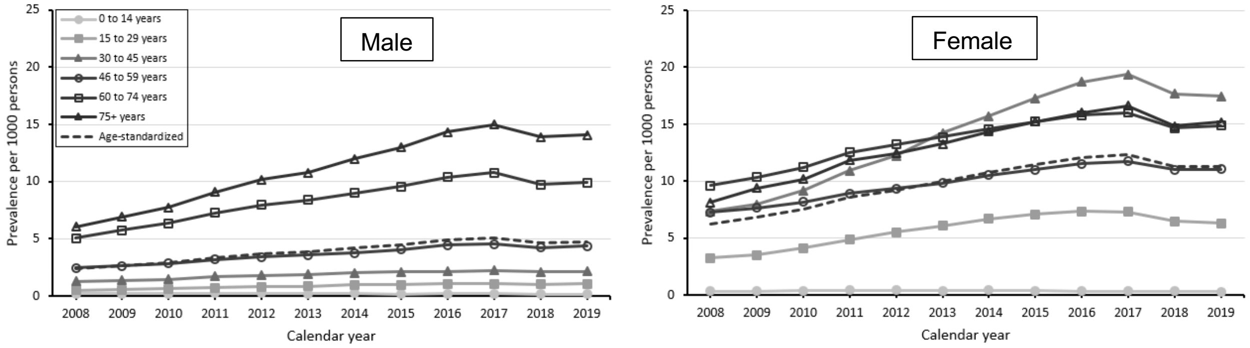

Figure 2.1: Age-standardized and age-specific prevalence of thyroid stimulating hormone measurement per 1,000 persons in 2019, stratified by healthcare utilization (quintiles of the number of outpatient treatment episodes in 2019) and sex

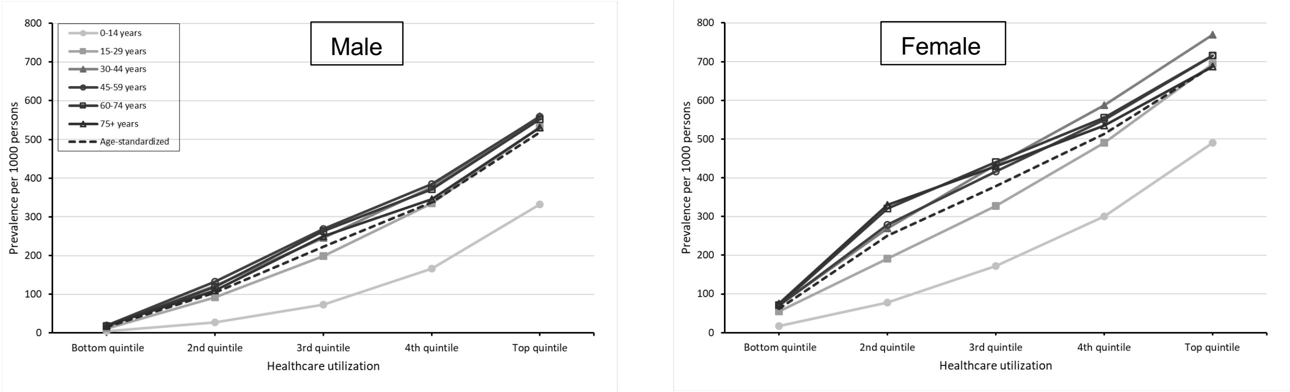

Figure 2.2: Age-standardized and age-specific prevalence of thyroid ultrasound per 1,000 persons in 2019, stratified by healthcare utilization and sex

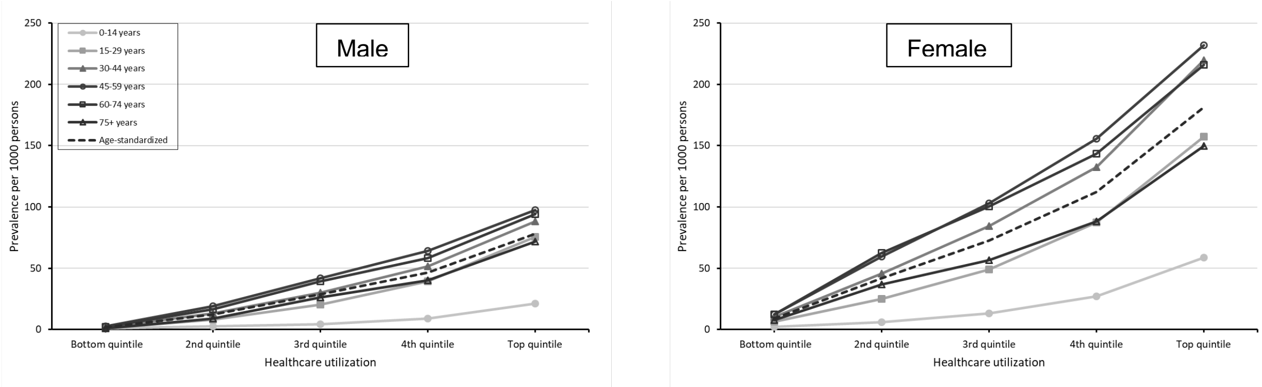

Figure 2.3: Age-standardized and age-specific prevalence of thyroid scintiscan per 1,000 persons in 2019, stratified by healthcare utilization and sex

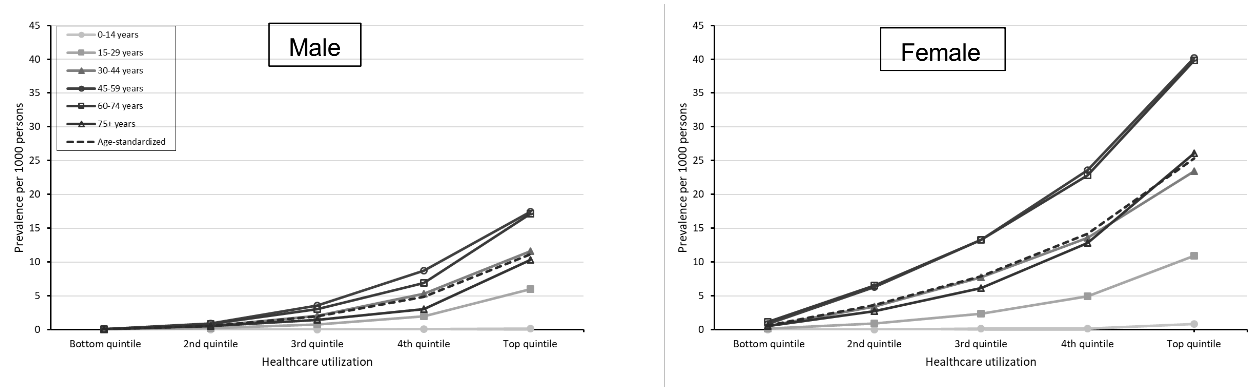

Figure 2.4: Age-standardized and age-specific prevalence of thyroid biopsy per 1,000 persons in 2019, stratified by healthcare utilization and sex

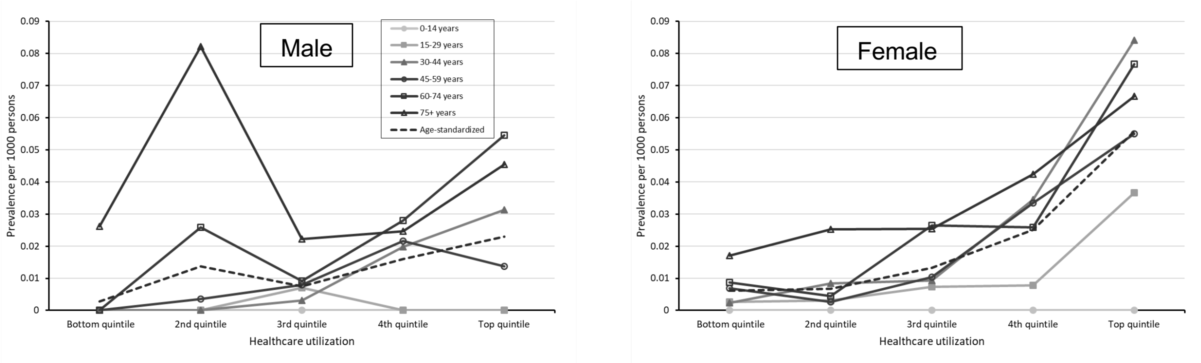

Figure 2.5: Age-standardized and age-specific prevalence of levothyroxine per 1,000 persons in 2019, stratified by healthcare utilization and sex

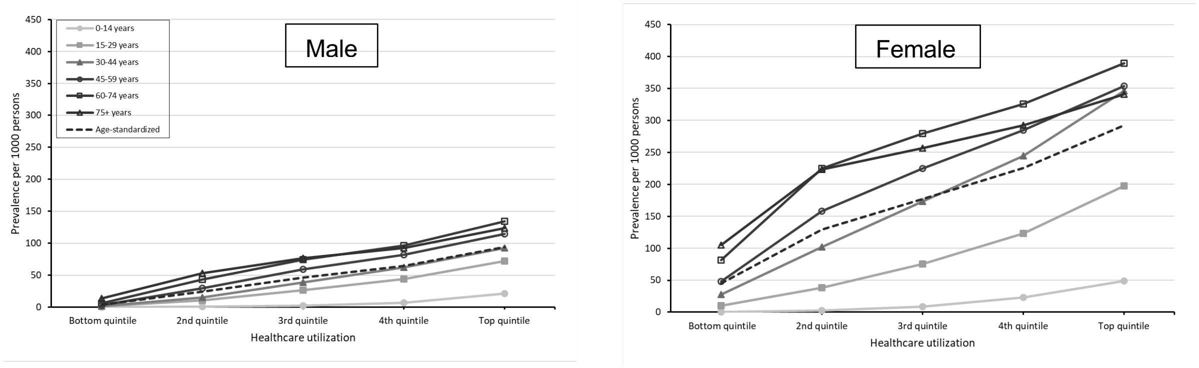

Figure 2.6: Age-standardized and age-specific prevalence of iodine per 1,000 persons in 2019, stratified by healthcare utilization and sex

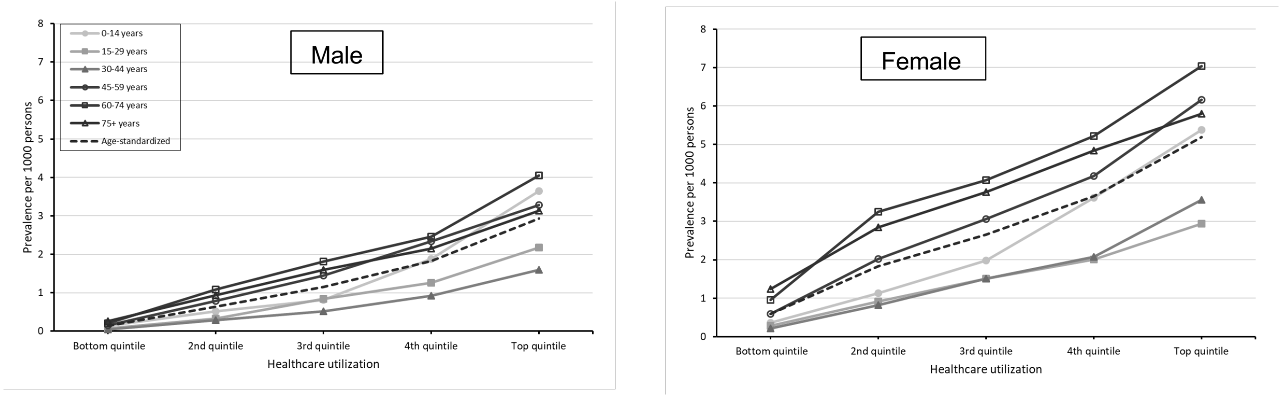

Figure 2.7: Age-standardized and age-specific prevalence of thyroidectomy per 1,000 persons in 2019, stratified by healthcare utilization and sex

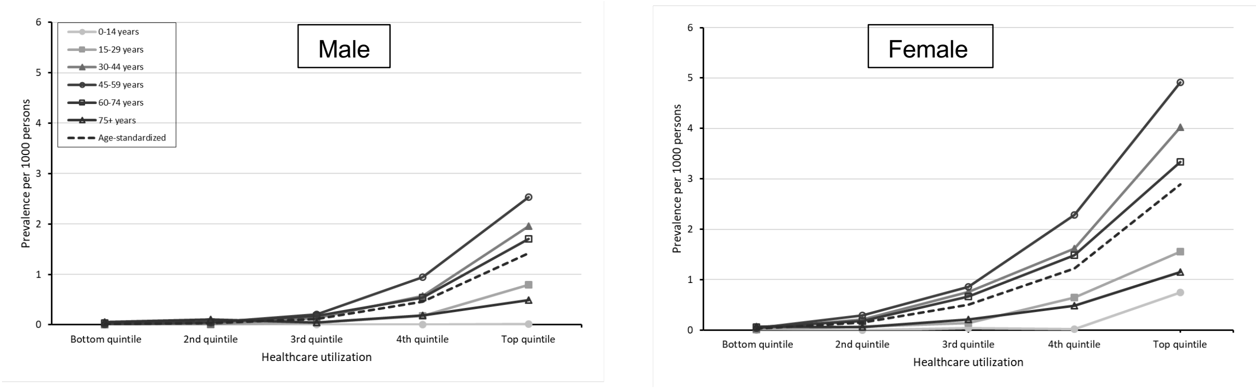

Figure 2.8: Age-standardized and age-specific prevalence of radioiodine therapy per 1,000 persons in 2019, stratified by healthcare utilization and sex

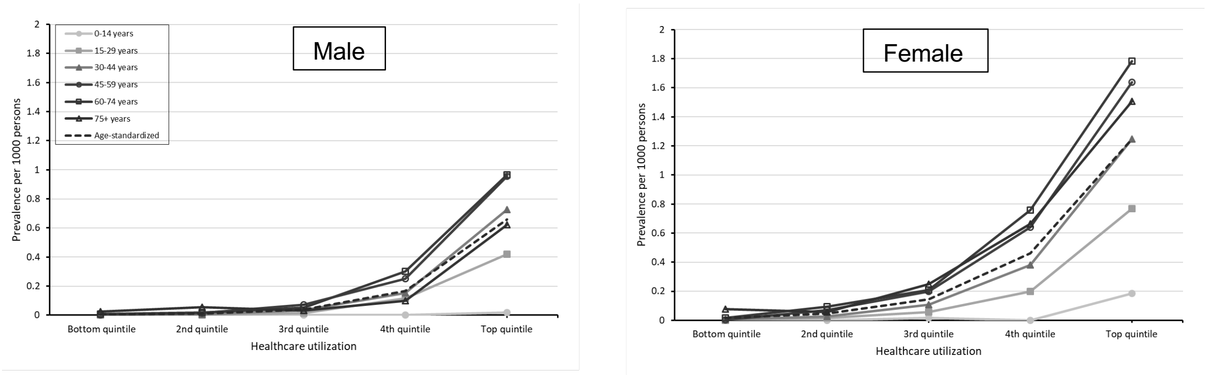

Figure 3: Annual age-standardized and age-specific prevalence of thyroid-stimulating hormone measurement per 1,000 persons from 2008 to 2019 (male and female combined)

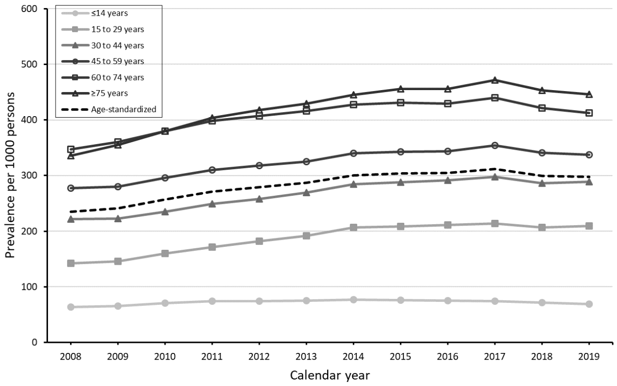

Figure 4: Annual age-standardized and age-specific prevalence of thyroid ultrasound per 1,000 persons from 2008 to 2019 (male and female combined)

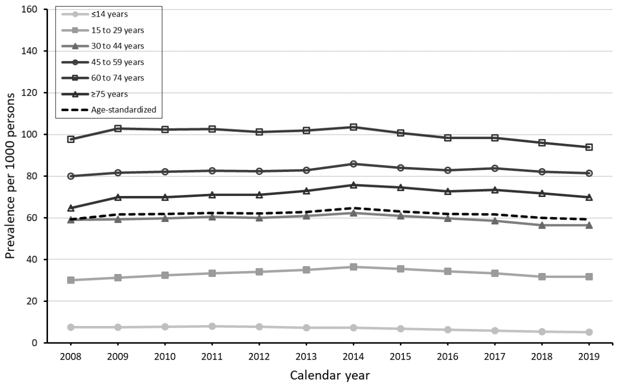

Figure 5: Annual age-standardized and age-specific prevalence of thyroid scintiscan per 1,000 persons from 2008 to 2019 (male and female combined)

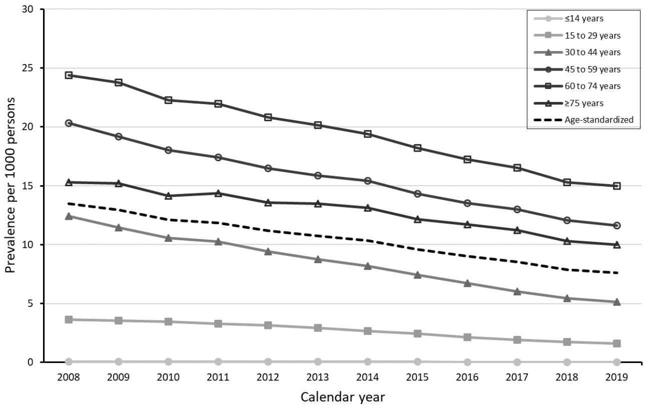

Figure 6: Annual age-standardized and age-specific prevalence of thyroid biopsy per 1,000 persons from 2008 to 2019 (male and female combined)

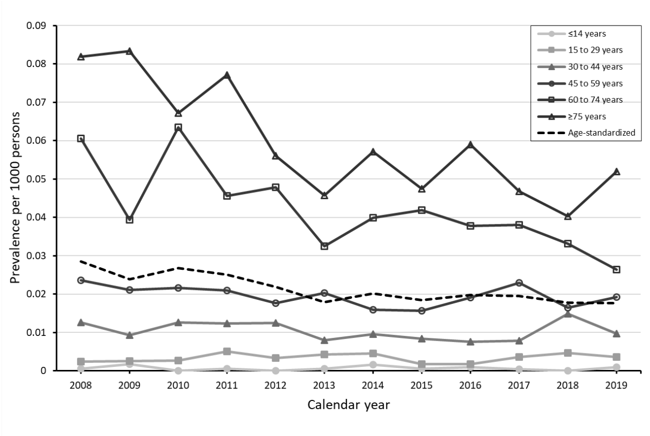

Figure 7: Annual age-standardized and age-specific prevalence of levothyroxine use per 1,000 persons from 2008 to 2019 (male and female combined)

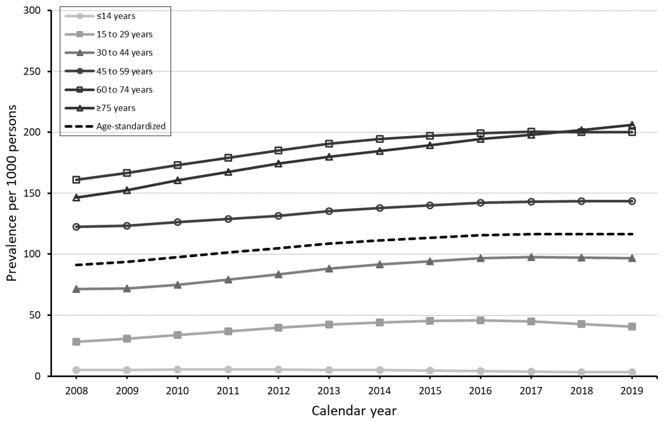

Figure 8: Annual age-standardized and age-specific prevalence of thionamide use per 1,000 persons from 2008 to 2019 (male and female combined)

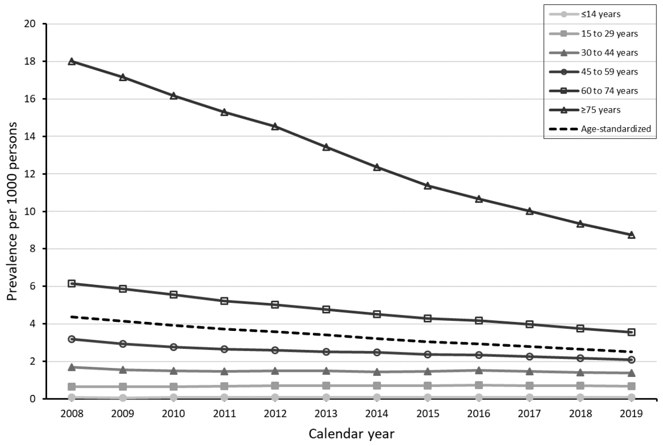

Figure 9: Annual age-standardized and age-specific prevalence of iodine use per 1,000 persons from 2008 to 2019 (male and female combined)

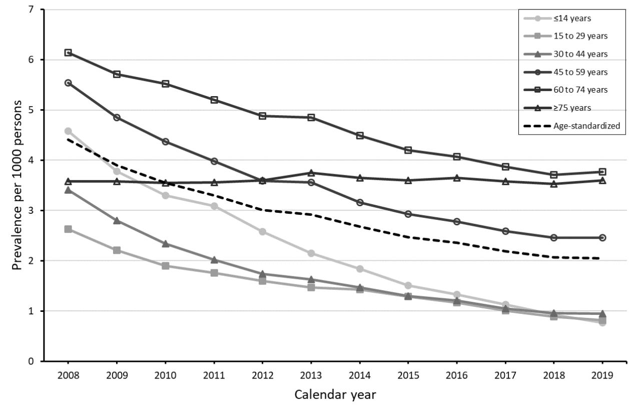

Figure 10: Annual age-standardized and age-specific prevalence of thyroid surgery per 1,000 persons from 2008 to 2019 (male and female combined)

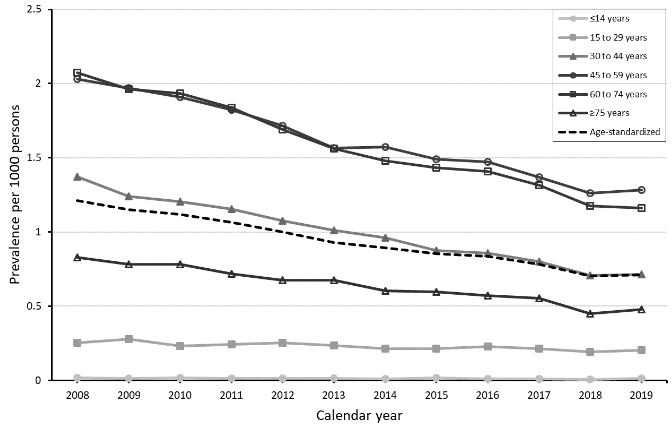

Figure 11: Annual age-standardized and age-specific prevalence of radioiodine therapy per 1,000 persons from 2008 to 2019 (male and female combined)

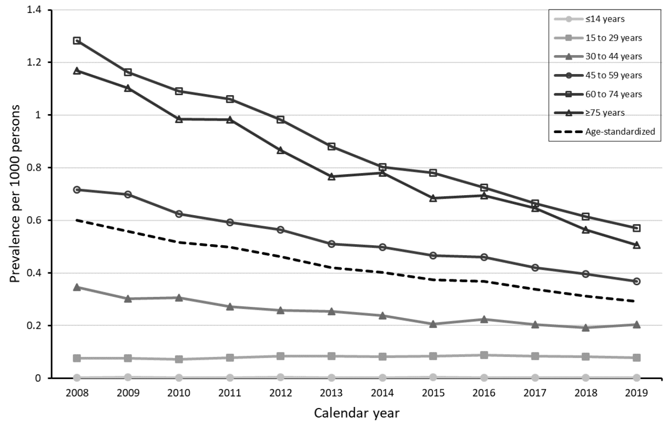

Supplement: Supplementary file 1 — Supplementary Material 1 [file 41598_2024_77896_MOESM1_ESM.pdf]
